# Development of antibacterial composite resin containing chitosan/fluoride microparticles as pit and fissure sealant to prevent caries

**DOI:** 10.1080/20002297.2021.2008615

**Published:** 2021-12-27

**Authors:** Chun-Cheng Lai, Chun-Pin Lin, Yin-Lin Wang

**Affiliations:** a Pediatric Dentistry, Far Eastern Memorial Hospital, Taipei, Taiwan; b Graduate Institute of Clinical Dentistry, School of Dentistry, National Taiwan University, Taipei, Taiwan; c College of Medicine, National Taiwan University Hospital, National Taiwan University, Taipei, Taiwan

**Keywords:** Fissure sealant, chitosan/fluoride microparticles, antibacterial, fluoride release, fluoride recharge

## Abstract

**Objective:**

Develop a fissure sealant containing chitosan/fluoride microparticles (C/F) with antibacterial, fluoride release and recharge ability.

**Materials and Methods:**

Chitosan/fluoride microparticles were synthesized and added to Bis-GMA as C/F. The experimental group comprised 0%, 2%, 4% C/F, with Clinpro^TM^ fissure sealant as control. Antibacterial activity was detected by Alamar Blue assay and colony-forming units (CFU). Biocompatibility was determined by WST-1 and LDH test. Curing depth, flowability, tensile strength and flexural strength were measured according to the ISO standard; microhardness by Vickers hardness test. Fluoride release and recharge were recorded through ionic chromatography. Statistical analysis was performed with an independent t-test, one-way and two-way ANOVA. P values less than 0.05 were considered significant.

**Results:**

2% and 4% C/F showed antibacterial ability with CFU ratios decreasing to 10% and 25% respectively (*P* < 0.01). Nonetheless, 4% C/F was concerned because biocompatibility revealed cytotoxicity compared to medium (*P* < 0.001). 2% C/F had superior mechanical properties to Clinpro^TM^ fissure sealant in terms of curing depth (*P* < 0.001), microhardness and tensile strength (*P* < 0.01). It had good fluoride release and recharge ability (*P* = 0.67).

**Conclusions:**

2% C/F could be an antibacterial sealant with good mechanical strength, fluoride release and recharge ability.

## Introduction

Dental caries is caused by a breakdown of tooth enamel that results from an imbalance between demineralization and remineralization of the tooth’s surface [1]. The occlusal surfaces are prone to attack by oral biofilm, a thin layer of bacteria, due to complex pits and fissures in which bacteria and food remnants can accumulate [2]. For this reason, dental sealants have been developed as a physical barrier bonded to the tooth surface and to protect teeth from destruction [3].

Occlusal fissures are complicated and easily attacked by bacteria, so fissure sealants with good sealing ability and lateral wall adaptation are needed [4]. Many commercial resin-based sealants are composed of dimethacrylate-based monomers, such as bisphenol A glycerolate dimethacrylate (Bis-GMA) or triethyleneglycol dimethacrylate (TEGDMA) with camphorquinone (CQ) and ethyl 4-dimethylaminobenzoate (EDMAB) as the most commonly used photo initiator. After an inorganic acid etches the occlusal surface, the monomers interact with each other and polymerization occurs, which enables the sealant to adhere firmly to the enamel surface [5]. Although these resin-based sealants have clinically acceptable retention rates, marginal leakages after the thermo-cycling process have been found in some laboratory studies [6,7].

Antibacterial substances have been considered as additions to fissure sealants to avoid secondary caries, such as bioglasses [8], nanoamorphous calcium phosphate [9], chlorhexidine-encapsulated nanotubes [10] or cationic nanocomposite containing silver bromide [11]. A systematic review assessed the antimicrobial effects of different antibacterial agents incorporated in resin-based dental sealants and revealed that those based on quaternary ammonium compounds stand out [12]. Therefore, Huang added dimethylaminododecyl methacrylate (DMADDM) to pit and fissure sealants and tried to achieve sustainable antibacterial effects [13]. Another antibacterial material frequently considered was that composed of fluoride [14–16]. The action of fluoride includes inhibition of demineralization, enhancement of remineralization, and bactericidal activity [17–20]. Most scientists have affirmative opinions on effects of decreased demineralization and increased remineralization. However, the bactericidal action is controversial. According to a hypothesis proposed by Li and Bowden [21], the bactericidal activity is due to the formation of hydrofluoric acid (HF) in the oral cavity and transported into cells. The pH value is altered, killing the bacteria. This explanation is paradoxical because the acid dissociation constant of HF is 3.14, and only 1% of fluoride converts to HF in the oral cavity, where the pH is 5. At present, a more convincing hypothesis is that fluoride has a repressive action on bacteria due to influence on enolase during glycolysis [22].

Several scientists have proposed adding chitosan, a natural antibacterial substance, to resin-based sealants, toothpaste, or mouthwash [23,24]. The chemical formula of chitosan is (1-4)-2-amino-2-deoxy-β-D-glucan. It originates from de-acetylation of chitin. When the degree of de-acetylation is more than 50%, the surface charge of chitosan becomes positive because of deprivation of an electron ion from the amino group. Positively charged chitosan molecules interfere with negatively charged residues on the bacterial surface. The wall of irregular bacteria can be broken and the contents are infiltrated. In this study, we would like to develop a fissure sealant with chitosan/fluoride microparticles (abbreviated as C/F), which has antibacterial, fluoride release and recharge capacities.

The hypothesis is that C/F has better antibacterial effect and mechanical properties than Clinpro^TM^ fissure sealant, a commercial product, and it also has fluoride release and recharge ability.

## Materials and methods

### Preparation of chitosan/fluoride microparticles and silanized silica nanoparticles

The synthesis of chitosan/fluoride microparticles was modified from the study of Niousha [25]. Chitosan (10 mg mL^−1^ 75–85% deacetylated, medium molecular weight, 448877, Sigma-Aldrich, St. Louis, MO) was dissolved in 500 mL distilled water containing acetic acid (5 mL 99.7%, 695092, Sigma-Aldrich, St. Louis, MO) on a magnetic stirrer (Corning PC-620D, Corning, NY) at 60°C for 5 h, and at room temperature for another 12 h. Sodium fluoride (NaF; 60 mg mL^−1^, 106441, Merck, Darmstadt, Germany) and tripolyphosphate (380 mg, 238503, Sigma-Aldrich, St. Louis, MO) were added to the solution and dissolved for 0.5 h, respectively. Particles were allowed to stabilize at room temperature overnight, centrifuged at 1,000 rpm, and collected after a freeze-drying process.

Ethanol (75 mL 97.2%, 24105, Sigma-Aldrich, St. Louis, MO) was prepared and the pH was adjusted to 3.5–4 with acetic acid. Nano Silica (15 g, CK-NS96, CHOKO CO., LTD, Taiwan) was added slowly to ethanol. Silanization was done with γ-methacryloxypropyltrimethoxysilane (0.75 g, OFS-6030, XIAMETER, Taiwan) on a magnetic stirrer at room temperature for 1 day. The solution was centrifuged at 4,000 rpm, collected, and dried at 50°C overnight.

### *Preparation of composite resin (see* Table 1)

C/F was prepared from chitosan/fluoride microparticles and a light-curable polymer matrix. This polymer matrix was prepared by mixing Bis-GMA, TEGDMA, EDMAB, and CQ in a weight ratio of 69:30:1:1 (Sigma-Aldrich, St. Louis, MO). The previously prepared silanized silica nanoparticles and C/F act as inorganic fillers, making up 20% of the composite resin. The polymer matrix was temporarily heated to 50°C, which enhanced the flowability and avoided trapped bubbles. Subsequently, the inorganic fillers were slowly added and stirred into matrix. The experimental composite resin was divided into three groups based on the composition of inorganic fillers: 1) 0% C/F and 20% silanized silica nanoparticles, abbreviated as 0% C/F; 2) 2% C/F and 18% silanized silica nanoparticles, abbreviated as 2% C/F; and 3) 4% C/F and 16% silanized silica nanoparticles, abbreviated as 4% C/F. Clinpro^TM^ fissure sealant (3 M ESPE, USA) was used as a control. Subsequent polymerization was carried out using an LED curing light with luminosity over 1,200 mw/cm^2^ and a wavelength of 460–510 nm (Motion LED-320D, Taipei, Taiwan).

**Table 1.** The composition of samples

	Components	(wt./wt. %)	Weight (g)
Control resin	Filler	20,000	0.600
	Bis-GMA, TEGDMA (7:3)	78,400	2,352
	Camphorquinone	0.800	0.024
	EDMAB	0.800	0.024
Experimental resin (2% /4%)	Filler	18,000/16,000	0.540/0.480
	CF	2,000/4,000	0.060/0.120
	Bis-GMA, TEGDMA (7:3)	78,400	2,352
	Camphorquinone	0.800	0.024
	EDMAB	0.800	0.024
Total		100,000	3,000

### Structure and size of particles

Fourier transforms infrared spectroscopy (FTIR) spectra were recorded on an FTIR spectrophotometer (JASCO FT/IR-4200, Tokyo, Japan) for the prepared, lyophilized chitosan/fluoride microparticles, Average particle size and distribution were determined by using a Dynamic Light Scattering (DLS)/Zeta Potential Analyzer (DLS Zetasizer Ultra, Malvern, UK).

### Bacterial strains and culture conditions

*Streptococcus mutans* (GS-5), a bacterium contributory to tooth decay, was used. Yeast extract (1%) was added to a brain heart infusion (BHI) broth for growth of *S. mutans*, and incubated for 48 h at 37°C under anaerobic conditions (10% H_2_, 10% CO_2_, and 80% N_2_). Growth was monitored by measuring optical density (OD) at 600 nm and colony forming units (CFU mL^−1^) every 2 h, until the growth curve advanced to exponential phase at 12 h. Serial dilution of the bacterial suspension to 1 × 10^6^-fold was needed to keep CFU mL^−1^ in the countable range from 30 to 300.

### Antibacterial ability

The compositions of experimental resin (0% C/F, 2% C/F, 4% C/F) and Clinpro^TM^ fissure sealant (n = 3) used in this study are listed in Table 1. Composite resin was put into a Teflon mold (6 mm diameter x 3 mm depth) and covered with a glass slide (0.15 mm thickness). Specimens were polymerized for 60 s, removed from the mold, and polished gently with 600 and 800 grit sandpaper sheets. Specimens were sterilized in ethylene oxide gas.

The inhibitory effect of C/F was detected with Alamar Blue assay and CFU mL^−1^. The experiment was performed in triplicate. First the standard curve was plotted by the ELISA reader (Hitachi TM-3000 tabletop SEM, Tokyo, Japan) with serial dilution of the bacterial suspension. The excitation wavelength was 530 nm, and the emission wavelength was 590 nm.

The samples were suspended with 125 μL of BHI broth containing *S. mutans* for 12 h anaerobically in the orbital shaking incubator (LM-400D, Yihder, Taipei, Taiwan), extracted and reacted with Alamar Blue agent for 3 h in the dark. The residual number of bacteria was calculated by the standard curve. We also counted CFU mL^−1^ using serial dilution from 10-fold to 10^6^-fold. A 100-μL aliquot of the bacterial suspension was spread onto BHI agar plates. The plates were incubated anaerobically for 48 h at 37°C and the number of total CFU mL^−1^ was determined.

Disks were immersed in half-strength Karnovsky’s solution (2% paraformaldehyde, 2.5% glutaraldehyde, pH 7.4) for 30 min. Fixed samples were dehydrated using 50, 75, 90, and 100% (v/v) graded ethanol, heat-dried at 37°C for 48 h, and coated with platinum by a touch-screen fully automatic sputter coater (Q150R Plus Rotary Pumped Coater, Quorum, Lewes, England). *S. mutans* cells were observed under a scanning electron microscope (SEM, Hitachi TM-3000 tabletop SEM, Tokyo Japan) with accelerating voltages of 10 KV using 2,000 x and 5,000 x magnification.

### Cell survival experiment

Five replicates were prepared for the biocompatibility of C/F. Cell survival was evaluated with 3T3 fibroblasts using a WST-1 test based on the ISO 10993–5 standard. The extraction solution was prepared according to the ISO 10993–12 standard. We added 1% L-glutamine, 1% sodium pyruvate, 1% non-essential amino acids solution, and 10% fetal bovine serum to Dulbecco’s modified Eagle medium (DMEM), and the cells were incubated at 37°C in a 5% CO_2_-humidified atmosphere for 3 days. The 3T3 cells were seeded in 96-well plates at a cell density of 1 × 10^4^ cells/well and incubated in a 5% CO_2_ atmosphere for 1 day. Then the DMEM was replaced with the extraction solution, which was diluted 10-fold before and after DMEM, respectively. Samples were incubated for 1, 3, and 5 days, with DMEM as control. After incubation, the plates were placed in an ELISA reader and OD was measured at 490 nm. The cells were also observed under an inverted microscope (SAGE Vision IVM-1A, SAGE Vision Co., Ltd., Taipei, Taiwan).

To determine cytotoxicity, 50 μL of the supernatant from each incubation period was aspirated and stored at −20°C. Next, we prepared a 50-μL standard solution for samples with released lactate dehydrogenase (LDH, 100 mm Tris-HCl buffer, pH 7.1, 15 mm NADH, 1.0 M pyruvate sodium salt) and total LDH (the same composition as for released LDH plus 10 μL 10% solution of Triton-X-100). The standard solution was incubated at 37°C for 30 min in the dark before measurement with the ELISA reader. Absorbance was set to 490 nm. Total LDH was designated as the high control group with maximum LDH release, and DMEM was designated as the low control group with spontaneous LDH release. Cell cytotoxicity was measured by Equation (1).

(1) $$Cytotoxicity(\%)=\frac{exp.value-lowcontrol}{highcontrol-lowcontrol}\times 100$$

### Mechanical properties

We selected 2% C/F to be compared with the mechanical strength of Clinpro^TM^ fissure sealant due to effective antibacterial effect and good biocompatibility. The parameters included curing depth, microhardness, diametral tensile strength, flexural strength, and flowability.

The curing depth was tested and repeated three times according to ISO 6874:2015 guidelines: fill a stainless-steel mold (4 mm diameter x 6 mm depth) with resin and cure in one direction for 30 s using an LED unit. The resin was released from the mold and the curing depth was recorded.

Microhardness was determined based on Vickers hardness test. We filled a Teflon mold (6 mm diameter x 3 mm depth) with either 2% C/F or Clinpro^TM^ fissure sealant. Following bidirectional irradiation with blue light for 20 s and subsequent demolding, the sample surfaces were wet polished with sandpaper from 400- to 1,000-grit. Microhardness was determined using a diamond indenter with a 0.05-kg load and a 30-s dwell time. Measurements were performed with a microhardness tester (HMV-2, Shimadzu, Kyoto, Japan) in six replicates.

Diametral tensile strength of six samples was measured according to the New American Dental Association Specification No. 27 method. Cylindrical samples were fabricated in a Teflon mold (6 mm diameter x 3 mm depth) and polymerized for 20 s. The six specimens were mounted vertically between disks of a universal testing machine (Instron 5566, Canton, MA) with crosshead speed 0.5 mm min^−1^ and measure the strength with Equation (2).

(2) $$(\sigma t)=\frac{2P}{\pi DT}$$

where σ_t_ is the tensile stress, P is the load at fracture, π is ratio of the circumference of a circle to its diameter, D is the diameter, and T is the thickness.

Flexural strength was measured according to the ISO 4049 guidelines using a Teflon mold (24 mm length x 2 mm width x 2 mm height) with the cross-sectional area 4 mm^2^: place the samples on a three-point bending test machine with a 20 mm distance between supports. The pressure at a 0.5 mm min^−1^ crosshead speed was exerted to measure the flexural strength from Equation (3).

(3) $$(\alpha )=\frac{3FL}{2bh^{2}}$$

where α is the flexural stress, F is the load (kN), L is the span length (cm), h is the sample thickness (cm), and b is the sample width (cm).

Flowability of samples was tested according to ISO 6876:2012 guidelines. Two glass slides (52 mm wide x 76 mm long) were prepared. A10-μL sample was placed on one glass slide and another slide was placed on top of it with 150 g force. After 1 min, the diameter of the spreading composite resin was measured and repeated three times.

### Fluoride release and recharge

Fluoride release and recharge ability of 2% C/F and Clinpro^TM^ fissure sealant were measured with ionic chromatography (883, Metrohm, Herisau, AR, Switzerland). Three samples were prepared for each material using a mold (6 mm diameter x 2 mm depth) and polymerized for 20 s. A batch method was used to measure the release of fluoride. Each material was placed in deionized (DI) water (5 mL), which was collected and replaced every 24 h for 6 days. A 0.22-μm filter was used to remove particles larger than 0.45 μm.

On day 7, disks were removed and sonication was applied (3 x 15 mL DI water for 10 min) to remove residual fluoride ions on the surface. Samples were placed in 5 mL 0.2% aqueous NaF, vibrated with a digital rotator (DSR2100P, Digisystem Laboratory Instruments Inc., Taipei, Taiwan) for 1 min, and placed in 5 mL DI water to measure fluoride release for another 3 days.

### Statistical analyses

Statistical results are presented as the mean ± standard deviation (SD). All P values are based on a 2-tailed statistical analysis, performed with one-way ANOVA for antibacterial ability, an independent sample t-test for mechanical properties, and two-way ANOVA for biocompatibility and fluoride release and recharge. Tukey’s test was used for multiple comparisons. P values less than 0.05 were considered statistically significant. Microsoft Excel 2010 (Microsoft Corporation) was used for all statistical calculations.

## Results

### Chemical structure and size of chitosan/fluoride microparticles

**Figure 1.**
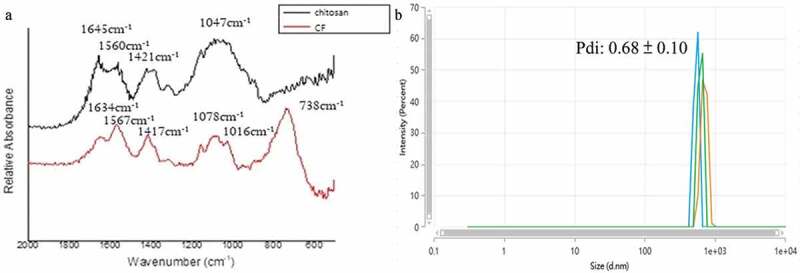
Chemical structure and size of chitosan/fluoride microparticles. (**A**) FTIR spectrum to detect specific functional group formed between NaF and chitosan (**B**) A representative graph of the light intensity versus the estimated particle diameter detected by DLS analyzer

The FTIR spectra of chitosan showed two peaks absorbing strongly at 1,421 and 1,560 cm^−1^, suspected to be the carbon–oxygen double bond, C = O, in the acetyl group(see Figure 1A). We noted some shift in the FTIR spectra of C/F, with the two peaks at 1,417 and 1,567 cm^−1^. In addition, a strong absorbance appeared at the peak of 738 cm^−1^, which we speculated to be the CF_3_ group. The average particle size of C/F was determined by DLS to be 629 nm. The polydispersity index (Pdi) was 0.68 (see Figure 1B).

### *Growth curve of* S. mutans

**Figure 2.**
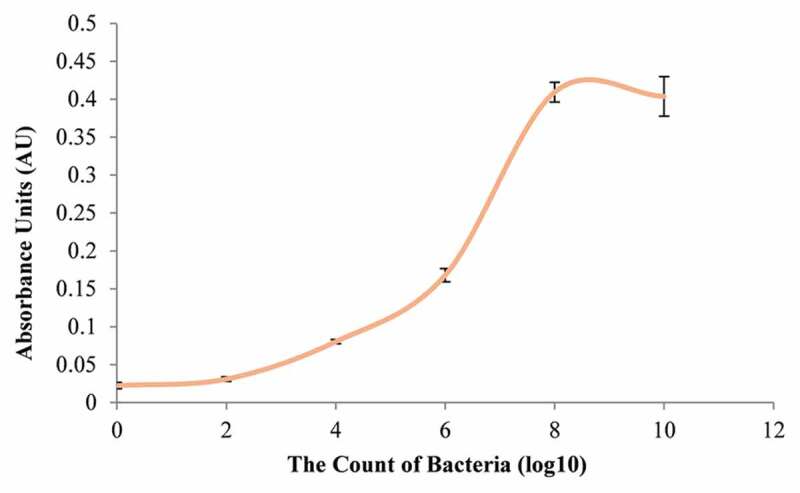
Growth curve of *S. mutans*. The bacteria cultured in a brain heart infusion (BHI) broth added with yeast extract (1%) for 48 h at 37°C

The growth curve of *S. mutans* was plotted at an initial pH of 7.0. Cells reached the late exponential/early stationary phase after 8 h of growth. This result demonstrated that the OD at 600 nm of growth was 0.022 in lag phase. *S. mutans* grew to mid-log phase after 4 h of incubation (OD = 0.080) and grew to early stationary phase after 8 h (OD = 0.410) (see Figure 2). The OD after 10 h was 0.404, which was not statistically different from the OD after 8 h (*P* = 0.74). In terms of CFU mL ^−1^, the values at 6 h, 8 h, and 10 h were 6.2 x 10^8^, 4.9 x 10^8^, and 6.5 × 10^8^ CFU mL^−1^, respectively (see supplementary Table A.1). The number of *S. mutans* at the early stationary stage was approximately 1 × 10^8^ CFU mL^−1^.

### Antibacterial ability

When the number of *S. mutans* was at 10^8^ CFU mL^−1^, the absorbance of fluorescence was 2638 AU, whereas at 10^6^ and 10^5^ CFU mL^−1^, the absorbance descended to 571 AU and 489 AU, respectively (see supplementary Figure A.1). The standard curve revealed a limit in the sensitivity of the Alamar Blue assay when the number of bacteria was less than 10^6^ CFU mL^−1^. The absorbance of fluorescence of pure bacterial suspension was 752 AU at late exponential phase (see supplementary Table B.1). All resins significantly inhibited the growth of *S. mutans* (*P* < 0.001). The OD of 0% C/F, 2% C/F, 4% C/F, and Clinpro^TM^ fissure sealant was 62%, 47%, 58%, and 67%, respectively, when compared to pure bacterial suspension. We found no statistical difference between resins (*P* = 0.19), which we suspect is due to the limited sensitivity of the Alamar Blue kit.

The CFU mL^−1^ of pure bacterial suspension was significantly higher than that of experimental resins (*P* < 0.001). In addition, antibacterial ability, presented as CFU ratio, differed between resins. The CFU ratio of 0% C/F, 2% C/F, 4% C/F, and Clinpro^TM^ fissure sealant was 54%, 10%, 25%, and 45%, respectively, when compared to pure bacterial suspension (see Table 2). The CFU ratio of 2% C/F and 4% C/F was significantly lower than 0% C/F and Clinpro^TM^ fissure sealant (*P* < 0.01). We found no difference between 2% C/F and 4% C/F (*P* = 0.15).

**Table 2.** The CFU ratio of C/F and the Clinpro^TM^ fissure sealant

	Pure suspension (n = 3)		0% /CF (n = 3)		2%/ CF (n = 3)		4% C/F (n = 3)		Clinpro^TM^ (n = 3)	
	M	SD	M	SD	M	SD	M	SD	M	SD
CFU Ratio	1.00^a^	0.00	0.54^b^	0.12	0.10^bc^	0.03	0.25^bc^	0.01	0.45^b^	0.10

^a-c^Different letters in the same column indicate significant statistical difference (*P* < 0.05, Tukey’s test)

**Figure 3.**
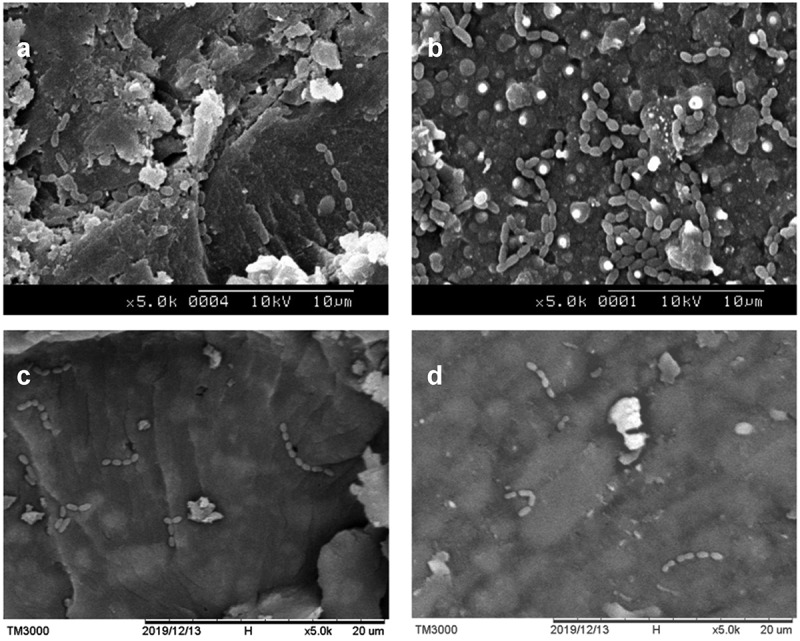
SEM images of bacteria-containing disks. **(A)** 0% C/F and **(B)** Clinpro^TM^ fissure sealant with poor antibacterial ability; **(C)** 2% C/F and **(D)** 4% C/F showed strong antibacterial effect against the GS-5 strain after 48 h of incubation. (magnification 5,000 x)

*S. mutans* presented as short chains of coccus-shaped bacteria. In the images of 0% C/F at 5,000 x magnification, some bacteria adhered to surface defects or around inorganic fillers (see Figure 3A). An abundance of *S. mutans* was found on the surface of Clinpro^TM^ fissure sealant, suggesting the limited antibacterial effect of fluoride (see Figure 3B). In comparison, scarce bacteria were found on the surface of 2% C/F (see Figure 3C) and 4% C/F (see Figure 3D), revealing strong antibacterial ability.

### Cell survival experiment

**Figure 4.**
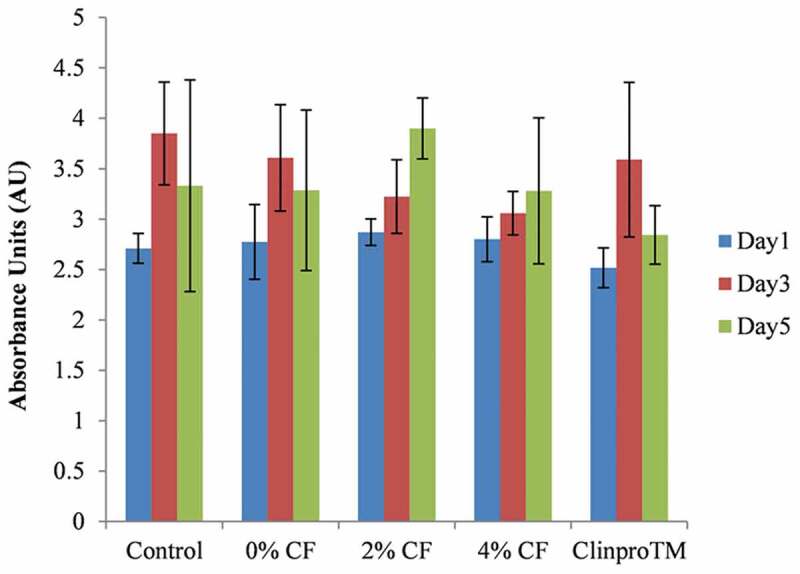
*In vitro* cytocompatibility of C/F. Quantification of cell viability on C/F compared to well culture plate and Clinpro^TM^ fissure sealant after 1, 3, and 5 days

After incubation in pure extraction solution for 1 day, the number of 3T3 fibroblasts in the treatments with 4% C/F and Clinpro^TM^ fissure sealant decreased markedly (see supplementary Figure B.1). The OD was 0.23 and 0.07 for these two treatments, respectively, compared to 1.96 for the control, a statistically significant difference (*P* < 0.001) (see supplementary Table C.1). The cells observed under a microscope also showed obvious necrosis. For this reason, the extraction solutions were diluted 10-fold to measure biocompatibility and cytotoxicity. After dilution, a WST-1 test (see Figure 4) and an LDH test (see Table 3) showed that the cells proliferated very quickly, and the cytotoxicity was negligible (*P =* 0.87). Because the extraction solution of 4% C/F may reduce cell proliferation and because no difference was noted in antibacterial ability between 2% C/F and 4% C/F, 2% C/F was chosen to compare the mechanical properties with the Clinpro^TM^ fissure sealant.

**Table 3.** Lactate dehydrogenase test to observe cell toxicity in diluted extraction solution

	High Control (n = 5)		Low Control (n = 5)		0% CF (n = 5)		2% CF (n = 5)		4% CF (n = 5)		Clinpro^TM^ (n = 5)	
	M	SD	M	SD	M	SD	M	SD	M	SD	M	SD
Day1(AU)	NA	NA	0.25	0.04	0.29	0.01	0.30	0.01	0.30	0.02	0.31	0.01
Day3(AU)	2.78	0.17	0.29	0.03	0.28	0.01	0.28	0.01	0.28	0.02	0.28	0.03
Day5(AU)	NA	NA	1.38	0.12	1.36	0.19	1.34	0.21	1.32	0.10	1.13	014

### *Mechanical strength (see* Table 4)

The curing depth of 2% C/F was higher than for the Clinpro^TM^ fissure sealant (5.93 vs 3.63 mm, *P* < 0.001). However, the average values were all greater than the ISO standard of 1.5 mm. Microhardness of 2% C/F was greater than with the Clinpro^TM^ fissure sealant (19.77 vs 16.70 kgfmm^−2^, *P* < 0.01), and diametral tensile strength of 2% C/F was also significantly higher (41.56 vs 29.14 Mpa, *P* < 0.01). Flowability of 2% C/F was lower than for the Clinpro^TM^ fissure sealant with a viscous property (8.83 vs 21.67 mm, *P* < 0.01). The flexural strength of 2% C/F and the Clinpro^TM^ fissure sealant was 73.18 and 68.57 Mpa, which was not statistically different (*P* = 0.72).

**Table 4.** The comparison of mechanical properties between 2% C/F and the Clinpro^TM^ fissure sealant

Materials	Curing depth (mm) (n = 3)		Microhardness (kgf mm^−2^) (n = 6)		Diametral tensile Strength (Mpa) (n = 6)		Flexural strength (Mpa) (n = 3)		Flowability (mm) (n = 3)	
	M	SD	M	SD	M	SD	M	SD	M	SD
Clinpro^TM^	3.63	0.32	16.70	1.29	29.14	5.24	68.58	15.24	21.67*	2.52
2% C/F	5.93*	0.12	19.77*	1.26	41.56*	5.10	73.18	13.57	8.83	0.82

*Significant at the 0.05 level (2-tailed)

#### Fluoride release and recharge

**Figure 5.**
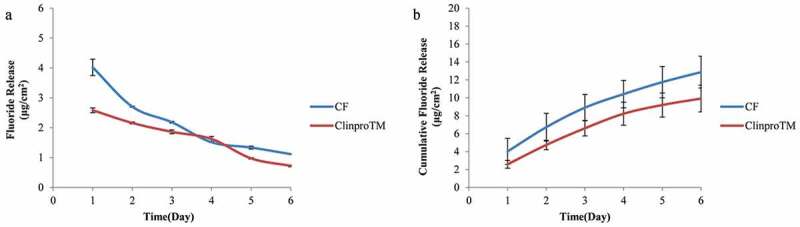
Amount of fluoride released per specimen surface area (μg/ cm^2^) from 2% C/F and Clinpro^TM^ fissure sealant **(A)** A representative graph of the releasing amount versus the time (day) **(B)** Cumulative fluoride release for 6 day

Two-way ANOVA showed differences in fluoride release ability *(P* < 0.001). From Tukey’s test, higher amounts of fluoride were released with 2% C/F compared to Clinpro^TM^ fissure sealant on day 1 and day 2 (see Figure 5A). The single-day release on day 3 was similar to that of Clinpro^TM^ fissure sealant, with a pattern of a relatively slow decrease over time. However, no significant difference in cumulative fluoride release was noted over 6 days (*P* = 0.09) (see Figure 5B). In terms of fluoride recharge ability, the cumulative fluoride release over 3 days was also not significantly different (*P* = 0.67) (see Table 5). The total amount of fluoride released after recharge was 4.59 μg cm^−2^ for 2% C/F and 5.16 μg cm^−2^ for the Clinpro^TM^ fissure sealant. From these results, we confirmed that 2% C/F exhibited fluoride release and recharge ability comparable to the Clinpro^TM^ fissure sealant.

**Table 5.** Fluoride recharge ability of 2% C/F and the Clinpro^TM^ fissure sealant

	Day1 (n = 3)		Day2 (n = 3)		Day3 (n = 3)		Total amount of release (n = 3)	
	M	SD	M	SD	M	SD	M	SD
2% C/F (μg cm^−2^)	1.92	0.81	1.67	0.57	1.01	0.41	4.59	1.78
Clinpro^TM^ (μg cm^−2^)	2.61	0.42	1.60	0.63	0.95	0.21	5.16	1.24

## Discussion

The purpose of this study was to develop a new light-curable antibacterial fissure sealant with fluoride release and recharge ability. We modified the structure of chitosan with NaF to form chitosan/fluoride microparticles. The preparation method was based on the study of Niousha [25]. In this study, the concentration of chitosan increased from 1 mg mL^−1^ to 10 mg mL^−1^ following the guidelines of Sigma-Aldrich. The concentration of NaF also increased from 12 mg mL^−1^ to 60 mg mL^−1^, in an effort to bind more fluoride ions to chitosan, enhancing its fluoride release ability. In Niousha’s study, the hydrodynamic radius was 219 nm and the Pdi was 0.45. In our study, the radius was 629 nm and the Pdi was 0.68. The larger particles and heterogeneous size distribution may be related to the increased amount of fluoride that we used, resulting in decrease of the zeta potential. Aggregation occurred, and led to increased size due to more neutral charges formed [26]

We chose 2% C/F and 4% C/F for our study. The reason to choose 2% was based on the study of Mahapoka [23]. The study revealed that 2% chitosan was needed to inhibit the growth of *S. mutans* because the decreasing amount in CFU mL^−1^ was 72.2%. When the chitosan content was lower than 2%, the decrease in CFU mL^−1^ was only 31.2–32.9%. We chose 4% C/F because 4% was the highest concentration among the studies reviewed [27,28]. Massouda [29] created antimicrobial extruded films by melt-processing chitosan with ethylene methyl acrylate copolymer ionomers and ethylene vinyl acetate copolymers. Log_10_ reductions of CFU mL^−1^ in films containing 4% chitosan after 24 h in a shake-flask test were near 2.

Interestingly, we found that 0% C/F had some antibacterial effects on *S. mutans*. According to the study of Lin [30], leachables from composite resin inhibited the growth of *S. mutans*. Through MTT and crystal violet assays, 500 μg/mL TEGDMA and 50 μg/mL Bis-GMA reduced 24-h metabolic activity and biofilm biomass. The number of cells with intact membranes decreased and the efficiency of acid production slowed.

Regarding biocompatibility and cytotoxicity, cell necrosis was found when 3T3 fibroblasts were incubated in pure extraction solution of 4% C/F and Clinpro^TM^ fissure sealant. Koulaouzidou [31] identified 1) organic components eluted from five resin dental sealants by using gas chromatography and mass spectrometry after 1 day and 40 days of storage, and 2) the effect of sealants on cell survival of cultured fibroblasts. The five dental sealants included BeautiSealant (SHOFU), Clinpro (3 M/ESPE), Conseal f (SDI), Grandio Seal (VOCO), and Helioseal Clear (Ivoclar/Vivadent). The study revealed a reduction of cell viability with Clinpro^TM^ fissure sealant because it eluted greater amounts of TEGDMA, indicating that TEGDMA could be primarily responsible for the damage of mitochondria and oxidative stress of cells.

The cytotoxicity of 4% C/F was suspected due to the negative influence of high fluoride concentration on the cells. The amount of fluoride release from 4% C/F was approximately 10 ppm, measured with ionic chromatography. Berry and Trillwood [32] explored the cytotoxicity of fluoride. HeLa cells and L mouse fibroblasts were cultivated in medium with 0.1 ppm, 1 ppm, and 10 ppm NaF for 7 days. The survival rate for HeLa cells and L mouse fibroblasts exposed to 0.1 ppm and 1 ppm was 82.7–86.8%, but decreased to 72.3% and 64.2% with 10 ppm. According to the ISO10993-5 guideline, if the cell survival rate decreases to 70%, cytotoxicity is a concern. The study concluded that this reduction of growth from such concentration was probably due to a decreased rate of cell division, not to immediate death. Oguro [33] proposed that, although the cell lines used in the studies were variable, 8–20 ppm of fluoride might inhibit the proliferation of cells incipiently, and the biological mechanism was shut down when the concentration increased to 40–60 ppm.

Proper mechanical properties are inevitable for pit and fissure sealants to endure various occlusal forces and wear in the mouth [34]. In the present study, the microhardness and diametral tensile strength of 2% C/F was significantly higher than for the Clinpro^TM^ fissure sealant. Thus, chitosan/fluoride microparticles would not cause the fracture of sealant material because of the acceptable values. The curing depth of 2% C/F was even deeper than for the Clinpro^TM^ fissure sealant. The result may be ascribed to the high transparency of resin monomer, nearly equal to the wavelength of light transmission, after impregnated with micro-sized chitosan [23]. Nevertheless, the flexural strength of 2% C/F was not different from the commercial product, because even if heating management was done before light curing, some air bubbles remained in the structure. The flowability was significantly lower when compared to the Clinpro^TM^ fissure sealant, perhaps due to a higher content of inorganic fillers, resulting in a viscous property.

In the section of fluoride release and recharge, we measured the concentration with the batch method by using ionic chromatography due to its high sensitivity [35,36]. The ion-selective electrode and the SPADNS method could also be used to measure the fluoride amount. The ion-selective electrode was a transducer that converted the motion of a specific ion into an electrical potential [37].The SPADNS method was used to measure the amount of fluoride ions by combining with zirconium in a red solution [38]. The colorless complex then bleached the red color in proportional to the fluoride concentration.

In this study, we proved the hypothesis that C/F has better antibacterial effect and mechanical properties than the Clinpro^TM^ fissure sealant, and it also has good fluoride release and recharge ability similar to the commercial product. However, some research methods could be improved. The sensitivity of the Alamar Blue assay was limited to detect bacteria lower than 1 × 10^6^ CFU mL^−1^; therefore, the antibacterial ability of C/F by the colorimetric method could not evidently be revealed. The crystal violet assay could perform better for the relationship between OD and bacterial number. In other aspects, the concentration of fluoride release and recharge of C/F was not higher than but as good as those of the Clinpro^TM^ fissure sealant, may be because only fluoride on the surface was measured, not the whole sample. Crushing of samples could be considered to measure the amount of fluoride release because the fissure sealant was under occlusal pressure in the oral environment.

## Conclusion

Chitosan/fluoride microparticles incorporated into dimethacrylate-based fissure sealant has been developed as a method to prevent caries. In this study, 2% chitosan/fluoride microparticles in resin sealant exhibited strong antibacterial property against *S. mutans*. It also showed good biocompatibility and superior mechanical properties, promising fluoride release and recharge ability.

## Supplementary Material

Supplemental Material
