# Supplementary material for: Development of antibacterial composite resin containing chitosan/fluoride microparticles as pit and fissure sealant to prevent caries
Source: J Oral Microbiol. 2021 Dec 27;14(1):2008615. doi: 10.1080/20002297.2021.2008615 (PMC8725701; doi:10.1080/20002297.2021.2008615)
Supplement: Supplemental Material [file ZJOM_A_2008615_SM1669.zip › Supplementary files/Table A1_clean.docx]

**Table A.1.** Growth curve for *S. mutans* calculated with CFU

| Time(h) | Colony Forming Units (CFU /mL x 10^7^) | | | Mean±SD |
| --- | --- | --- | --- | --- |
| 6 | 80 | 44 | 61 | 62±18  49±16  65±26 |
| 8 | 67 | 38 | 41 |  |
| 10 | 95 | 45 | 56 |  |
