# Supplementary material for: Development of antibacterial composite resin containing chitosan/fluoride microparticles as pit and fissure sealant to prevent caries
Source: J Oral Microbiol. 2021 Dec 27;14(1):2008615. doi: 10.1080/20002297.2021.2008615 (PMC8725701; doi:10.1080/20002297.2021.2008615)
Supplement: Supplemental Material [file ZJOM_A_2008615_SM1669.zip › Supplementary files/Table B1_clean.docx]

**Table B.1.** The inhibitory effect of C/F detected with Alamar Blue assay

|  | Control  (n=3) | | 0% C/F  (n=3) | | 2% C/F  (n=3) | | 4% C/F  (n=3) | | Clinpro^TM^ (n=3) | |
| --- | --- | --- | --- | --- | --- | --- | --- | --- | --- | --- |
|  | M | SD | M | SD | M | SD | M | SD | M | SD |
| OD | 752^a^ | 35 | 464^b^ | 96 | 351^b^ | 49 | 433^b^ | 55 | 505^b^ | 103 |
| Ratio | 100% | | 62% | | 47% | | 58% | | 67% | |

^ab^: Different letters in the same column indicate significant statistical difference (*P*< 0.05, Tukey’s test)
