# Supplementary material for: Development of antibacterial composite resin containing chitosan/fluoride microparticles as pit and fissure sealant to prevent caries
Source: J Oral Microbiol. 2021 Dec 27;14(1):2008615. doi: 10.1080/20002297.2021.2008615 (PMC8725701; doi:10.1080/20002297.2021.2008615)
Supplement: Supplemental Material [file ZJOM_A_2008615_SM1669.zip › Supplementary files/Table C1_clean.docx]

**Table C.1.** WST-1 test to evaluate cell survival in pure extraction solution for 1 day

|  | Control  (n=5) | | | 0% C/F  (n=5) | | 2% C/F  (n=5) | | 4% C/F  (n=5) | | Clinpro^TM^  (n=5) | |
| --- | --- | --- | --- | --- | --- | --- | --- | --- | --- | --- | --- |
|  | M | | SD | M | SD | M | SD | M | SD | M | SD |
| OD (AU) | 1.96^a^ | 0.21 | | 2.19^a^ | 0.20 | 1.74^a^ | 0.31 | 0.23^b^ | 0.29 | 0.07^b^ | 0.15 |

^ab^: Different letter in the same row represents statistically different means (P < 0.05) by Tukey’s test.

Absorbance units: abbreviated as AU
