# Supplementary figures and images for: Development of antibacterial composite resin containing chitosan/fluoride microparticles as pit and fissure sealant to prevent caries
Source: J Oral Microbiol. 2021 Dec 27;14(1):2008615. doi: 10.1080/20002297.2021.2008615 (PMC8725701; doi:10.1080/20002297.2021.2008615)

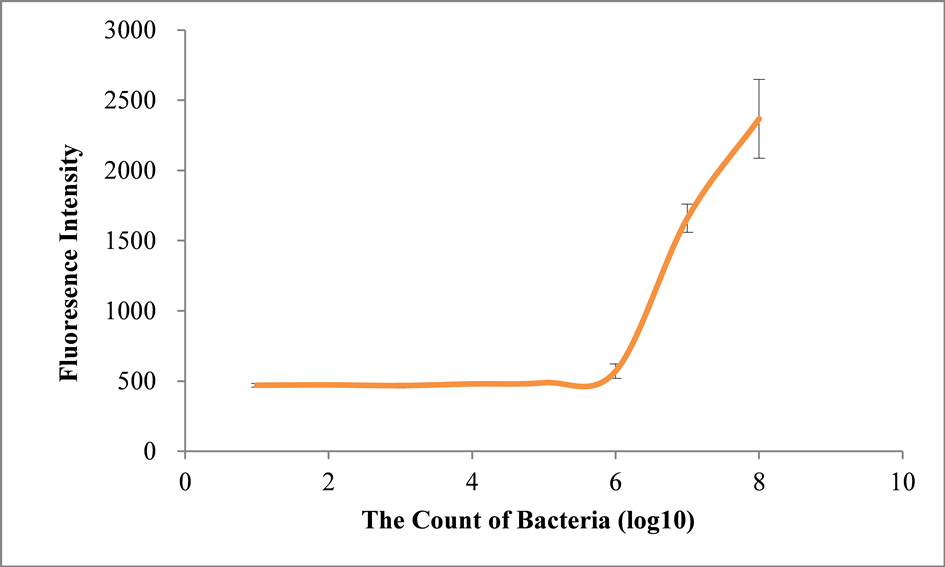

Supplement: Supplemental Material [file ZJOM_A_2008615_SM1669.zip › Supplementary files/Fig A1.tif]

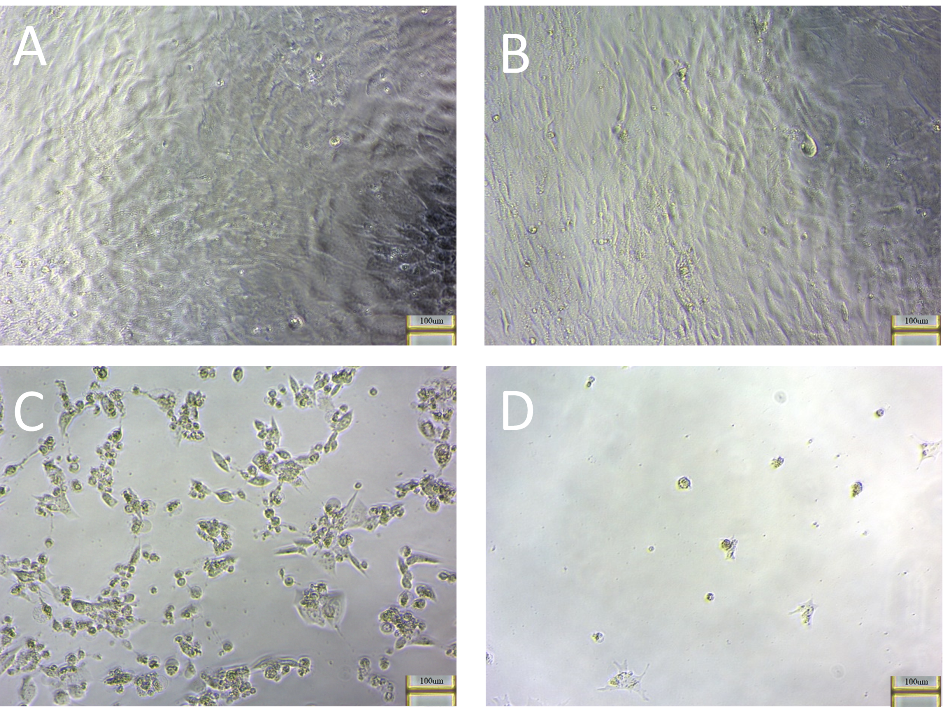

Supplement: Supplemental Material [file ZJOM_A_2008615_SM1669.zip › Supplementary files/Fig B1.tif]
